# Association of platelet-to-white blood cell ratio and platelet-to-neutrophil ratio with the risk of fatal stroke occurrence in middle-aged to older Chinese

**DOI:** 10.1186/s12877-022-03134-z

**Published:** 2022-05-17

**Authors:** Zhi-bing Hu, Qiong-qiong Zhong, Ze-xiong Lu, Feng Zhu

**Affiliations:** 1 Department of Internal Medicine and Central Laboratory, Guangzhou Twelfth People’s Hospital, Guangzhou, China; 2 Department of Public Health and Preventive Medicine, School of Medicine, Jinan University, Jinan, China; 3 Department of Internal Medicine, Sanya Central Hospital, Sanya, China

**Keywords:** White blood cell, Platelet, Neutrophil, Stroke, Ischaemic, Haemorrhagic

## Abstract

**Background:**

White blood cell (WBC) and neutrophil (NEUT) counts, which are commonly inflammatory markers, have been related to an increased risk of fatal stroke. However, it is unclear whether platelet-to-white blood cell ratio (PWR) and platelet-to-neutrophil ratio (PNR) are related to the risk of fatal stroke in middle-aged to older populations.

**Method:**

In total, 27,811 participants without a stroke history at baseline were included and followed up for a mean of 14.3 years (standard deviation = 3.2), and 838 stroke deaths were recorded. The Cox proportional hazards regression was used to assess the relationships between the PWR and the PNR and the risk of fatal strokes.

**Results:**

Compared to the 1^st^ quartile, an increased risk of fatal all stroke showed among the participants in the highest quartiles of both the WBC (adjusted hazard ratio (aHR) = 1.35, 95% confidence interval (CI) 1.09–1.66) and the NEUT (aHR = 1.45, 95% CI 1.18–1.79). The restricted cubic splines showed decreased trends in associations of the PWR and the PNR with the risk of fatal all stroke. A decreased risk of fatal all stroke showed in those with the highest quartiles for both the PWR (aHR = 0.73, 95% CI 0.53–1.00) and the PNR (aHR = 0.74, 95% CI 0.54–1.01). The participants with the 2^nd^, the 3^rd^ and the 4^th^ change quartiles for the PWR and the PNR had weak decreasing trends for the risk of fatal all stroke, compared to those in the 1^st^ change quartile, and the significant associations were observed in those with an increase of 20% for the PWR with the risk of fatal haemarragic stroke (aHR = 0.47, 95% CI 0.22–0.95) and a decrease of 20% for the PNR with the risk of fatal all stroke (aHR = 1.33, 95% CI 0.99–1.79), compared to those with stable dynamic changes.

**Conclusions:**

Higher neutrophil count and platelet-to-neutrophil ratio were associated with a contrary risk of fatal stroke, with an increased for the former and a decreased for the later. A potentially chronic inflammation should be paid close attention to stroke occurrence in relatively healthy middle-aged to older populations.

**Supplementary Information:**

The online version contains supplementary material available at 10.1186/s12877-022-03134-z.

## Background

Stroke, a major public health problem, has become a leading cause of deaths in China [1]. It is classified mainly as ischaemic and haemorrhagic stroke. A series of risk factors such as hypertension, diabetes and smoking have been known as main risk factors in stroke [2–6], and were closely related to a chronic inflammation [7]. Atherosclerosis, an inflammatory disease [8, 9], plays an important role in stroke pathophysiology. The WBC acts positively in atherosclerotic thrombosis [10], and it has been related to an increased risk of fatal stroke [11, 12]; the NEUT releases its extracellular traps (NETs) and activates endothelial cells and the platelets (PLTs) in atherosclerotic plaque rupture or erosion [13, 14]. Additionally, the PLTs aggravated inflammation, promoted atherosclerosis [15], and led acute ischemic events involving thrombotic and hemorrhagic diseases [16, 17].

The platelet-to-white blood cell ratio (PWR) has been linked to an independently mortality risk in patients with acute exacerbation of chronic liver failure[18] or undergoing radical cystectomy [19], and it was related to a 90-day disability or death in acute ischemic stroke [20]. Similarly, the platelet-to-neutrophil ratio (PNR) was related to a hospitalization or a long-term mortality in the patients with infective endocarditis [21], and was an independent risk factor for ischemic stroke [22]. However, there are few studies so far in systematic addressing the relationships between the PWR and the PNR and risks of fatal stroke and its subgroups in a general community population. In this study, we based on the Guangzhou Biobank cohort study (GBCS) to investigate systematically the associations of PWR and PNR with the risks of fatal all stroke, fatal ischaemic stroke and fatal haemorrhagic stroke in a relatively healthy middle-aged to older population.

## Methods

### Participants

All participants were recruited from a population of permanent residents aged 50 years or above in Guangzhou in southern China. Details of the GBCS have been reported previously [23]. The baseline(from September 1^st^, 2003 to February 28^th^, 2008) and follow up information included a face to face computer-assisted interview by trained nurses on lifestyle [24], the family and personal medical history and assessment of anthropometrics, blood pressure and laboratory tests. Each participant had been made an appointment in advance to ensure good health and was able to go to the designated place, and was able to sit and rest for at least half an hour before sampling and examination.

### Exposure indicators

Blood cell counts were performed with a cell counter (KX-21, Sysmex, Japan) in Guangzhou Twelfth People’s Hospital [25]. The PWR and the PNR were calculated respectively from the PLT and the WBC, the PLT and the NEUT. Fasting glucose, cholesterol, triglycerides, liver and kidney function and high sensitivity C-reactive protein (hs-CRP) were measured by an analyzer (Cobas c-311, Roche, Switzerland). The laboratory performs internal and external quality control procedures according to the China Association of Laboratory Quality Control.

### Study outcomes

Information on underlying causes of death up to April 13^th^ 2021 was obtained mostly via record linkage with the Guangzhou Centers for Disease Control and Prevention (GZCDC). Due to no other information for stroke severity, infarct volume, site of lesion and infectious complications as previous work [25], fatal stroke occurrence was chosen as only one outcome of this study. Death causes were coded according to the 10^th^ revision of the International Classification of Diseases (ICD) as follows: I60 ~ I69 for stroke; I60.0 ~ I62.9 and I69.0 ~ I69.2 for haemorrhagic stroke; I63.0 ~ I63.9 and I69.3 for ischaemic stroke; and the other codes for unclassified stroke. The death certificates were verified by the GZCDC as part of their quality assurance program by cross-checking past medical history and conducting verbal autopsy by 5 senior clinicians from Guangzhou Twelfth People’s Hospital, the Universities of Hong Kong, China and Birmingham, UK.

### Potential confounders

To examine the extent to which baseline factors in relation to the risks of fatal all stroke, fatal ischaemic stroke and fatal haemorrhagic stroke, we defined potential confounders based on the *P *value < 0.05 in quartiles of PWR or quartiles of PNR for risk factors, and a series of factors in different models were included, according to our previous work [25]. Model 1 was a crude hazard ratio model without an adjustment for any confounders. Model 2 contained a multivariate adjustment for factors including sex, age, smoking (never, former and current), alcohol consumption (never, former and current), International Physical Activity Questionnaire-assessed physical activity (inactive, moderate and active),body mass index (BMI, defined as weight in kg ÷ height in m^2^), self-rated health, hypertension, diabetes, dyslipidaemia, cancer, genitourinary disease (including nephropathy, prostatic disease, and gynecologic diseases), chest disease (including COPD, chronic bronchitis, emphysema, asthma, tuberculosis, and pneumonia) and platelet count. Model 3 included hs-CRP as a competing confounder in addition to confounders in model 2.

### Statistical analysis

A series of variables, including the WBC, the NEUT, the PLT, the PWR and the PNR, were respectively classified by quartiles: the1^st^ quartile (< 5.3*10^9/L), the 2^nd^ quartile (5.3–6.1*10^9/L), the 3^rd^ quartile (6.2–7.2*10^9/L) and the 4^th^quartile (> 7.2*10^9/L) for the WBCs; the1^st^ quartile (< 3.0*10^9/L), the 2^nd^ quartile (3.0–3.6*10^9/L), the 3^rd^quartile (3.7–4.4*10^9/L) and the 4^th^quartile (> 4.5*10^9/L) for the NEUTs; the1^st^ quartile (< 190*10^9/L), the 2^nd^ quartile (191–223*10^9/L), the 3^rd^ quartile (224–260*10^9/L) and the 4^th^quartile (> 260*10^9/L) for the PLTs; the 1^st^ quartile (≤ 30), the 2^nd^ quartile (30.01–36.11), the 3^rd^ quartile (36.12–43.38) and the 4^th^ quartile (≥ 43.39) for the PWRs; the 1^st^ quartile (≤ 48.64), the 2^nd^ quartile (48.65–61.11), the 3^rd^ quartile (61.12–76.25) and the 4^th^ quartile (≥ 76.25) for the PNRs. The distributions of PWR and PNR quartiles showed great ranges in which several extreme values were mainly included in the 1^st^ and the 4^th^ quartiles, because these values combined by other blood cell counts are not abnormal or missing in the corresponding individuals, although the PWR and the PNR were assessed as continuous parameters using a restricted cubic spline curve model (RCS) with 3 knots at the 10^th^, the 50^th^, and the 90^th^ percentiles, based on the smoothness of curves, the avoidance of reduction of accuracy caused by over fitting, and the easiness of explaining the relRationship between continuous variables and outcomes. Continuous variables were described by the mean ± standard deviation, and categorical variables were described by frequency and percentage. The PWR and PNR changes were calculated with the data from two times exposure period (the baseline (from September 2003 to February 2008) and the 1st follow-up (from March 2008 to December 2012)): Values of PWR and PN changes = [(PWR(PNR) _follow up_—PWR(PNR) _baseline_) ÷ PWR(PNR) _baseline_] × 100%. The chi-square test and Fisher’s exact test were used for categorical variables, and analysis of variance (ANOVA) and the Kruskal–Wallis test were used for continuous variables. Sensitivity analyses were conducted in which model 2 and model 3 was repeated with a further adjustment for hs-CRP. All analyses were performed using STATA (Version 14.0; StataCorp LP, College Station, TX, USA). All *p* values were 2 sided, and statistical significance was defined as *p* < 0.05; *p* values for trends in models were calculated as ordinal scores from the 2^nd^, the 3^rd^ and the 4^th^ quartiles when taking the 1^st^ as reference. All methods were performed in accordance with the Declaration of Helsinki.

## Results

### Baseline characteristics

**Fig. 1:**
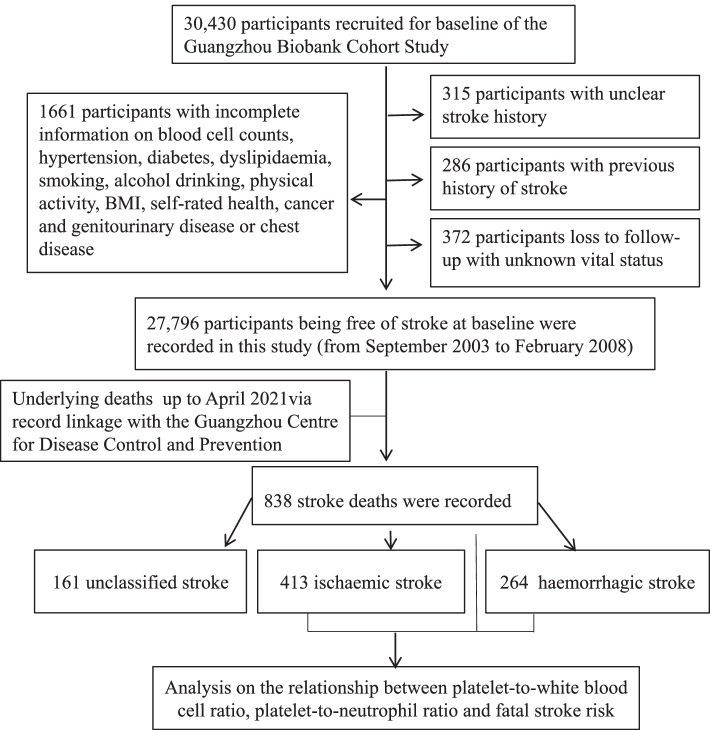
Flow diagram of participants selected for the analysis of this study

In total, 30,430 participants were screened, and 2,634 participants were excluded, including 286 because of a previous history of stroke, 315 because of an unclear stroke history, 372 because of loss to follow-up with unknown vital status, and 1,661 because of incomplete information on the WBC, the NEUT, the LYM and platelet counts, hypertension, diabetes, dyslipidaemia, smoking, alcohol consumption, physical activity, BMI, self-rated health, cancer, genitourinary disease or chest disease. Eventually a total of 27,796 participants at baseline were included, and 838 stroke deaths (413 ischaemic, 264 haemorrhagic and 161 unclassified) were recorded after a mean follow-up time of 14.3 (standard deviation = 3.2) years with 399,116 person-years in this study (Fig. 1).

The baseline characteristics are presented in Table 1. Compared to those in the 1^st^ quartile, the participants in the highest PWR and the PNR included more women, were younger, and had more dyslipidaemia, active physical activity, genitourinary disease. These subjects were less likely to have BMI ≥ 28 kg/m^2^, had lower hypertensive, and had less current smoking and alcohol drinking, and chest disease and diabetes. For the 1^st^ follow-up characteristics, the participants in the highest PWR and PNR included more men, were younger, and had more current smoking. These subjects were less of BMI ≥ 28 kg/m^2^, had lower active physical activity, hypertension, and had less cancer and chest disease, compared to those in the 1^st^ quartile (Supplementary Table 1).

**Table 1:** Baseline characteristics by the PWR and the PNR quartiles of participants in the GBCS (*n* = 27,796)

	Quartiles of PWR					Quartiles of PNR				
Characteristic	the 1^st^ (≤ 30)	the 2^nd^ (30.01–36.11)	the 3^rd^ (36.12–43.38)	the 4^th^ (≥43.39)	*P*	the 1^st^ (≤ 48.64)	the 2^nd^ (48.65–61.11)	the 3^rd^ (61.12–76.25)	the 4^th^ (≥76.25)	*P*
Number, n	7020	6872	6955	6949		6948	6843	6959	6946	
Age (years)	63.6 ± 7.0	62.5 ± 7.0	61.6 ± 7.0	60.3 ± 6.8	< 0.001	63.8 ± 7.1	62.6 ± 7.1	61.5 ± 7.0	60.1 ± 6.7	< 0.001
Sex, male (%)	3060 (43.6)	2029 (29.5)	1516 (21.8)	1021 (14.7)	< 0.001	3036 (43.7)	2110 (30.4)	1466 (21.1)	1014 (14.6)	< 0.001
Hypertension, n (%)	2257 (32.2)	2039 (29.7)	1908 (27.4)	1609 (23.2)	< 0.001	2298 (33.1)	2037 (29.3)	1932 (27.8)	1546 (22.3)	< 0.001
Diabetes, n (% )	1290 (18.4)	930 (13.5)	844 (12.1)	564 (8.1)	< 0.001	1280 (18.4)	958 (13.8)	822 (11.8)	568 (8.2)	< 0.001
Dyslipidaemia, n (%)	5571 (79.4)	5751 (83.7)	5837 (83.9)	5853 (84.2)	< 0.001	5485 (78.9)	5760 (83.0)	5871 (84.4)	5896 (84.9)	< 0.001
Smoking, n (%)					< 0.001					< 0.001
never	4857 (69.2)	5500 (80.0)	5913 (85.0)	6244 (89.8)		4780 (68.8)	5543 (79.8)	5946 (85.4)	6245 (89.9)	
ever	916 (13.0)	664 (9.7)	558 (8.0)	381 (5.5)		942 (13.6)	688 (9.9)	520 (7.5)	369 (5.3)	
current	1247 (17.8)	708 (10.3)	484 (7.0)	324 (4.7)		1226 (17.6)	712 (10.3)	493 (7.1)	332 (4.8)	
Alcohol drinking, n (%)					< 0.001					< 0.001
never	4772 (68.0)	4836 (70.4)	4965 (71.4)	4955 (71.3)		4760 (68.5)	4828 (69.5)	4939 (71.0)	5001 (72.0)	
ever	206 (2.9)	168 (2.4)	142 (2.0)	124 (1.8)		225 (3.2)	156 (2.2)	127 (1.8)	132 (1.9)	
current	2042 (29.1)	1868 (27.2)	1848 (26.6)	1870 (26.9)		1963 (28.3)	1959 (28.3)	1893 (27.2)	1813 (26.1)	
Body mass index, kg/m^2^					< 0.001					< 0.001
<18.5	266 (3.8)	288 (4.2)	302 (4.3)	390 (5.6)		285 (4.1)	308 (4.4)	298 (4.3)	355 (5.1)	
18.5 – 23.9	3258 (46.4)	3290 (47.9)	3532 (50.8)	3859 (55.6)		3320 (47.8)	3272 (47.1)	3480 (50.0)	3867 (55.7)	
24 – 27.9	2643 (37.6)	2531 (36.8)	2466 (35.5)	2212 (31.8)		2521 (36.3)	2604 (37.5)	2508 (36.0)	2219 (31.9)	
≥28	853 (12.2)	763 (11.1)	655 (9.4)	488 (7.0)		822 (11.8)	759 (10.9)	673 (9.7)	505 (7.3)	
Physical activity, n (%)					< 0.001					< 0.001
inactive	555 (7.9)	523 (7.6)	560 (8.1)	617 (8.9)		547 (7.9)	522 (7.5)	546 (7.8)	640 (9.2)	
moderate	2965 (42.2)	2873 (41.8)	2813 (40.4)	2685 (38.6)		2965 (42.7)	2939 (42.3)	2789 (40.1)	2643 (38.1)	
active	3500 (49.9)	3476 (50.6)	3582 (51.5)	3647 (52.5)		3436 (49.5)	3482 (50.2)	3624 (52.1)	3663 (52.7)	
Self-rated health, n (%)	5751 (81.9)	5670 (83.0)	5795 (83.3)	5714 (82.2)	0.097	5651 (81.3)	5798 (83.5)	5753 (82.7)	5764 (83.0)	0.006
(good/very good)										
Cancer, n (%)	123 (1.8)	123 (1.8)	157 (2.3)	136 (2.0)	0.12	109 (1.6)	133 (1.9)	151 (2.2)	146 (2.1)	0.04
GD, n (% )	1735 (24.7)	1770 (25.8)	1915 (27.5)	1982 (28.5)	< 0.001	1702 (24.5)	1832 (26.4)	1882 (27.0)	1986 (28.6)	< 0.001
Chest disease, n (%)	1155 (16.5)	1017 (14.8)	1011 (14.5)	1030 (14.8)	0.006	1123 (16.2)	1027 (14.8)	1027 (14.8)	1036 (14.9)	0.06
WBC, *10^9/L	7.5 ± 1.7	6.6 ± 1.3	6.0 ± 1.6	5.3 ± 1.1	< 0.001	7.6 ± 1.7	6.6 ± 1.2	6.0 ± 1.2	5.2 ± 1.0	< 0.001
NEUT, *10^9/L	4.7 ± 1.5	4.0 ± 1.3	3.6 ± 1.1	3.0 ± 0.9	< 0.001	5.0 ± 1.4	4.0 ± 0.9	3.5 ± 0.8	2.8 ± 0.7	< 0.001
PLT, *10^9/L	185.8 ± 46.3	218.2 ± 42.9	237.5 ± 46.0	268.0 ± 61.0	< 0.001	193.7 ± 50.7	220.8 ± 48.1	236.2 ± 51.1	258.3 ± 60.7	< 0.001
hs-CRP, mg/L	3.8 ± 3.1	3.5 ± 2.8	3.4 ± 2.8	3.3 ± 2.6	< 0.001	4.1 ± 3.2	3.5 ± 2.7	3.4 ± 2.7	3.1 ± 2.6	< 0.001
All stroke	295 (4.2)	215 (3.1)	188 (2.7)	140 (2.0)	< 0.001	315 (4.5)	210 (3.0)	186 (2.7)	127 (1.8)	< 0.001
Ischaemic stroke	146 (2.1)	116 (1.7)	88 (1.3)	63 (0.9)	< 0.001	167 (2.5)	102 (1.5)	83 (1.2)	61 (0.9)	< 0.001
Haemorrhagic stroke	99 (1.5)	58 (0.9)	62 (0.9)	45 (0.7)	< 0.001	95 (1.4)	62 (0.9)	66 (1.0)	41 (0.6)	< 0.001
Unclassified stroke	50 (0.7)	41 (0.6)	38 (0.6)	32 (0.5)	0.22	53 (0.8)	46 (0.7)	37 (0.5)	25 (0.4)	0.009

Hypertension: systolic blood pressure, ≥ 140 mmHg, or diastolic blood pressure, ≤ 90 mmHg, medication and diagnosis; diabetes: fasting blood glucose ≥ 7 mmol/L, medication or diagnosis; dyslipidaemia: total cholesterol ≥ 5.2 mmol/L, triglyceride ≥ 1.7 mmol/L, low density lipoprotein ≥ 3.4 mmol/L, high density lipoprotein < 1.0 mmol/L, medication and diagnosis

*WBC* white blood cell count, *hs-CRP* high sensitivity C-reactive protein, *NEUT* neutrophil, *Platelet* PLT, *PWR* platelet to white blood cell ratio, *PNR* platelet to neutrophil ratio, *GD* Genitourinary disease (including nephropathy, prostatic disease, and gynecologic diseases), *chest disease* including COPD, chronic bronchitis, emphysema, asthma, tuberculosis, and pneumonia

### The WBC, the NEUT and the PLT in relation to the risk of fatal stroke

We observed firstly that the participants in the highest WBC quartile had an increased risk of fatal all stroke (aHR = 1.35, 95% CI 1.09–1.66, *P* = 0.005), compared to those in the 1^st^ WBC quartile; and those in the 2^nd^, the 3^rd^ and the 4^th^ WBC quartiles had an increased risk trend in fatal all stroke (*P* < 0.001) and fatal ischaemic stroke (*P* = 0.002), respectively; The NEUTs had similar results for fatal all stroke (aHR = 1.45, 95% CI 1.18–1.79, *P* < 0.001) and fatal ischaemic stroke (aHR = 1.58, 95% CI 1.17–2.12, *P* = 0.03), respectively, However, no other significant relationships were observed between the PLT and the risk of fatal strokes besides an increased risk for fatal unclassified stroke (aHR = 1.72, 95% CI 1.11–2.65, *P* = 0.01) (Supplementary Table 2).

### The PWR and the PNR in relation to the risk of fatal stroke in the RCS model

**Fig. 2:**
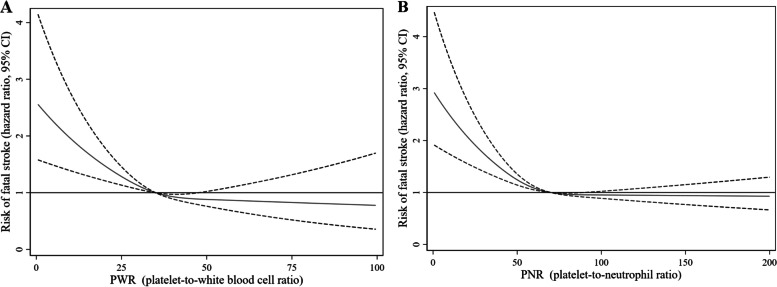
Associations of platelet-to-white blood cell ratio and the platelet-to-neutrophil ratio with the risk of fatal stroke in the restricted cubic spline curves model in the Guangzhou Biobank Cohort Study followed up for a mean 14.3 years. The solid blue line is the multivariable adjusted hazard ratio, with dashed lines showing 95% confidence intervals with three knots. A multivariate model was used, adjusted for sex, age, diabetes, hypertension, dyslipidaemia, smoking, alcohol consumption, physical activity, body mass index, self-rated health, cancer, genitourinary diseases, chest disease and platelet count

The RCS showed nonlinear relationships between the PWR and the PNR and the risk of fatal all stroke after adjustments for potential confounders. Higher levels of the PWR and the PNR were associated with a decreased risk of fatal all stroke, and the cutoff values were 35 for the PWR and 74 for the PNR (Fig. 2).

### The PWR in relation to the risk of fatal stroke

After adjustment for a series of factors and compared to those in the 1^st^ quartile, no significant associations of the PWR with the risks of fatal strokes were observed, although very weak decreasing trends for risks of fatal all stroke and fatal ischemic stroke were found among the participants in the 2^nd^, the 3^rd^and the 4^th^ PNR quartiles (the left side of Table 2). Such trends were strengthened, and the highest PWR quartile was related to a decreased risk of fatal ischemic stroke (aHR = 0.73, 95% CI 0.53–1.00, *P* = 0.05) among those without a history of relative cardiovascular diseases (CVD) at baseline and further adjustment for hs-CRP (the left side of Table 3).

**Table 2:** Associations of PWR and PNR with the risk of fatal stroke occurrence in the GBCS (*n* = 27,796)

	Quartiles of PWR				Quartiles of PNR			
	the 1^st^ (≤ 30)	the 2^nd^ (30.01–36.11)	the 3^rd^ (36.12–43.38)	the 4^th^ (≥ 43.39)	the 1^st^ (≤ 48.64)	the 2^nd^ (48.65–61.11)	the 3^rd^ (61.12–76.25)	the 4^th^ (≥76.25)
***All stroke***								
Model 1 (HR, 95% CI)	1.00	0.71 (0.60–0.85), *P* < 0.001	0.61 (0.51–0.74), *P* < 0.001	0.46 (0.38–0.56), *P* < 0.001	1.00	0.64 (0.54–0.76), *P* < 0.001	0.56 (0.47–0.67), *P* < 0.001	0.38 (0.31–0.47), *P* < 0.001
Model 2 (HR, 95% CI)	1.00	0.89 (0.74–1.06), *P* = 0.19	0.89 (0.74–1.07), *P* = 0.22	0.85 (0.69–1.05), *P* = 0.14	1.00	0.82 (0.68–0.97), *P* = 0.02	0.84 (0.70–1.01), *P* = 0.06	0.76 (0.61–0.94), *P* = 0.01
P for trend	0.38				0.03			
***Ischaemic stroke***								
Model 1 (HR, 95% CI)	1.00	0.77 (0.61–1.00), *P* = 0.04	0.58 (0.44–0.75), *P* < 0.001	0.42 (0.31–0.56), *P* < 0.001	1.00	0.59 (0.46–0.75), *P* < 0.001	0.47 (0.36–0.61), *P* < 0.001	0.34 (0.26–0.47), *P* < 0.001
Model 2 (HR, 95% CI)	1.00	0.99 (0.77–1.26), *P* = 0.93	0.88 (0.67–1.15), *P* = 0.35	0.84 (0.62–1.14), *P* = 0.26	1.00	0.77 (0.60–0.98), *P* = 0.04	0.74 (0.57–0.97), *P* = 0.03	0.74 (0.55–1.01), *P* = 0.05
*P* for trend	0.60				0.06			
***Haemorrhagic stroke***								
Model 1 (HR, 95% CI)	1.00	0.57 (0.41–0.79), *P* = 0.001	0.60 (0.44–0.82), *P* = 0.002	0.43 (0.30–0.62), *P* < 0.001	1.00	0.62 (0.45–0.86), *P* = 0.004	0.65 (0.47–0.89), *P* = 0.007	0.40 (0.28–0.58), *P* < 0.001
Model 2 (HR, 95% CI)	1.00	0.69 (0.50–0.95), *P* = 0.02	0.80 (0.58–1.11), *P* = 0.18	0.72 (0.49–1.03), *P* = 0.07	1.00	0.76 (0.55–1.05), *P* = 0.09	0.91 (0.66–1.26), *P* = 0.57	0.71 (0.48–1.04), *P* = 0.08
*P* for trend	0.10				0.21			
***Unclassified stroke***								
Model 1 (HR, 95% CI)	1.00	0.80 (0.53–1.21), *P* = 0.29	0.73 (0.48–1.11), *P* = 0.14	0.61 (0.39–0.96), *P* = 0.03	1.00	0.83 (0.56–0.1.23), *P* = 0.34	0.65 (0.43–0.99), *P* = 0.04	0.44 (0.27–0.71), *P* = 0.001
Model 2 (HR, 95% CI)	1.00	1.01 (0.67–1.53), *P* = 0.97	1.07 (0.70–1.64), *P* = 0.76	1.17 (0.74–1.86), *P* = 0.50	1.00	1.05 (0.71–1.57), *P* = 0.80	0.99 (0.65–1.53), *P* = 0.98	0.89 (0.55–1.47), *P* = 0.67
*P* for trend	0.91				0.94			

*PWR* platelet to white blood cell ratio, *PNR* platelet to neutrophil ratio, *model 1* a crude hazard ratio model without adjustment for confounders, *model 2* a multivariate model adjusted for age, sex, diabetes, hypertension, dyslipidaemia, smoking, alcohol consumption, physical activity, body mass index, self-rated health, cancer, genitourinary disease (nephropathy, prostatic disease, gynecologic diseases) and chest disease (COPD, chronic bronchitis, emphysema, asthma, tuberculosis, and pneumonia), and platelet count

**Table 3:** Associations of PWR and PNR with the risk of fatal stroke among the participants without CVD at baseline and further hs-CRP adjustment (*n* = 10,990)

		Quartiles of PWR				Quartiles of PNR		
	the 1^st^ (≤ 30)	the 2^nd^ (30.01–36.11)	the 3^rd^ (36.12–43.38)	the 4^th^ (≥ 43.39)	the 1^st^ (≤ 48.64)	the 2^nd^ (48.65–61.11)	the 3^rd^ (61.12–76.25)	the 4^th^ (≥ 76.25)
***All stroke***								
Model 1 (HR, 95% CI)	1.00	0.83 (0.66–1.06), *P* = 0.13	0.74 (0.57–0.94), *P* = 0.02	0.44 (0.32–0.60), *P* < 0.001	1.00	0.72(0.57–0.91), *P* = 0.007	0.60 (0.47–0.78), *P* < 0.001	0.42 (0.31–0.57), *P* < 0.001
Model 3 (HR, 95% CI)	1.00	0.98 (0.77–1.25), *P* = 0.88	0.97 (0.75–1.25), *P* = 0.82	0.73 (0.53–1.00), *P* = 0.05	1.00	0.86 (0.68–1.09), *P* = 0.22	0.83 (0.64–1.08), *P* = 0.18	0.74 (0.54–1.01),*P* = 0.06
* P* for trend	0.23				0.23			
***Ischaemic stroke***								
Model 1 (HR, 95% CI)	1.00	0.92 (0.66–1.28), *P* = 0.61	0.77 (0.54–1.10), *P* = 0.15	0.38 (0.24–0.61), *P* < 0.001	1.00	0.69 (0.49–0.96), *P* = 0.03	0.54 (0.37–0.78), *P* = 0.001	0.38 (0.25–0.59), *P* < 0.001
Model 3 (HR, 95% CI)	1.00	1.09 (0.78–1.53), *P* = 0.60	1.04 (0.72–1.49), *P* = 0.83	0.66 (0.41–1.08), *P* = 0.09	1.00	0.82 (0.59–1.15),*P* = 0.25	0.76 (0.52–1.11),*P* = 0.15	0.68 (0.43–1.07), *P* = 0.09
* P* for trend	0.22				0.29			
***Haemorrhagic stroke***								
Model 1 (HR, 95% CI)	1.00	0.65 (0.42–1.02), *P* = 0.06	0.72 (0.46–1.13), *P* = 0.15	0.52 (0.31–0.87), *P* = 0.01	1.00	0.65 (0.41–1.03), P = 0.06	0.86 (0.56–1.33), P = 0.50	0.52 (0.31–0.87), *P* = 0.01
Model 3 (HR, 95% CI)	1.00	0.75 (0.48–1.18), *P* = 0.21	0.91 (0.58–1.42), *P* = 0.67	0.79 (0.46–1.34), *P* = 0.38	1.00	0.77 (0.48–1.23), *P* = 0.27	1.16 (0.74–1.81), *P* = 0.52	0.85 (0.49–1.47), *P* = 0.56
* P* for trend	0.61				0.37			
***Unclassified stroke***								
Model 1 (HR, 95% CI)	1.00	0.90 (0.54–1.52), *P* = 0.71	0.66 (0.37–1.18), *P* = 0.16	0.43 (0.22–0.87), *P* = 0.02	1.00	0.88 (0.54–1.43), *P* = 0.60	0.38 (0.19–0.73), *P* = 0.004	0.38 (0.19–0.76), *P* = 0.006
Model 3 (HR, 95% CI)	1.00	1.07 (0.64–1.82), *P* = 0.79	0.90 (0.50–1.62), *P* = 0.62	0.74 (0.36–1.51), *P* = 0.41	1.00	1.07 (0.65–1.75), *P* = 0.80	0.55 (0.28–1.08), *P* = 0.08	0.71 (0.35–1.44), *P* = 0.34
* P* for trend	0.76				0.21			

*PWR* platelet to white blood cell ratio, *PNR* platelet to neutrophil ratio, *hs-CRP*: high sensitivity C-reactive protein, *CVD*: relative cardiovascular diseases, *model 1*: a crude hazard ratio model without adjustment for confounders, *model 3*: a multivariate model adjusted for age, sex, diabetes, hypertension, dyslipidaemia, smoking, alcohol consumption, physical activity, body mass index, self-rated health, cancer, genitourinary disease (nephropathy, prostatic disease, and gynecologic diseases), chest disease (COPD, chronic bronchitis, emphysema, asthma, tuberculosis, and pneumonia), platelet count, and hs-CRP

### The PNR in relation to the risk of fatal stroke

After adjustment for a series of factors and compared to those in the 1^st^ quartile, the participants in the 4^th^ PNR quartile were related to a decreased risk of fatal all stroke (aHR = 0.76, 95% CI 0.61–0.94, *P* = 0.03) and fatal ischemic stroke (aHR = 0.74, 95% CI 0.55–1.01, *P* = 0.06), respectively; the participants in the 2^nd^, the 3^rd^ and the 4^th^ PNR quartiles had a weak decreasing trends for risks of fatal all stroke (*P* = 0.03) and fatal ischemic stroke (*P* = 0.06) (the right side of Table 2). However, such trends were weakened, and no significant associations were found among those without CVD at baseline and further adjustment for hs-CRP, besides a weak decreased risk of fatal all stroke (aHR = 0.74, 95% CI 0.54–1.01) in those with the highest PNR quartile (the right side of Table 3).

### The PWR and PNR changes in relation to the risk of fatal stroke

The basic characteristics of the participants at the 1^st^ follow-up are shown in Supplementary table 1. The participants with a PWR gain (> 20%) had more men, higher proportions of former and current smokers, BMIs from 18.5 to 23.9 kg/m^2^and higher PLT counts; lower proportions of physical activity, BMIs ≥ 28 kg/m^2^, hypertension, chest disease and cancer; and lower WBC and NEUT counts (all *P* < 0.05), compared to those with a stable PWR (from − 20% to 20%).

**Fig. 3:**
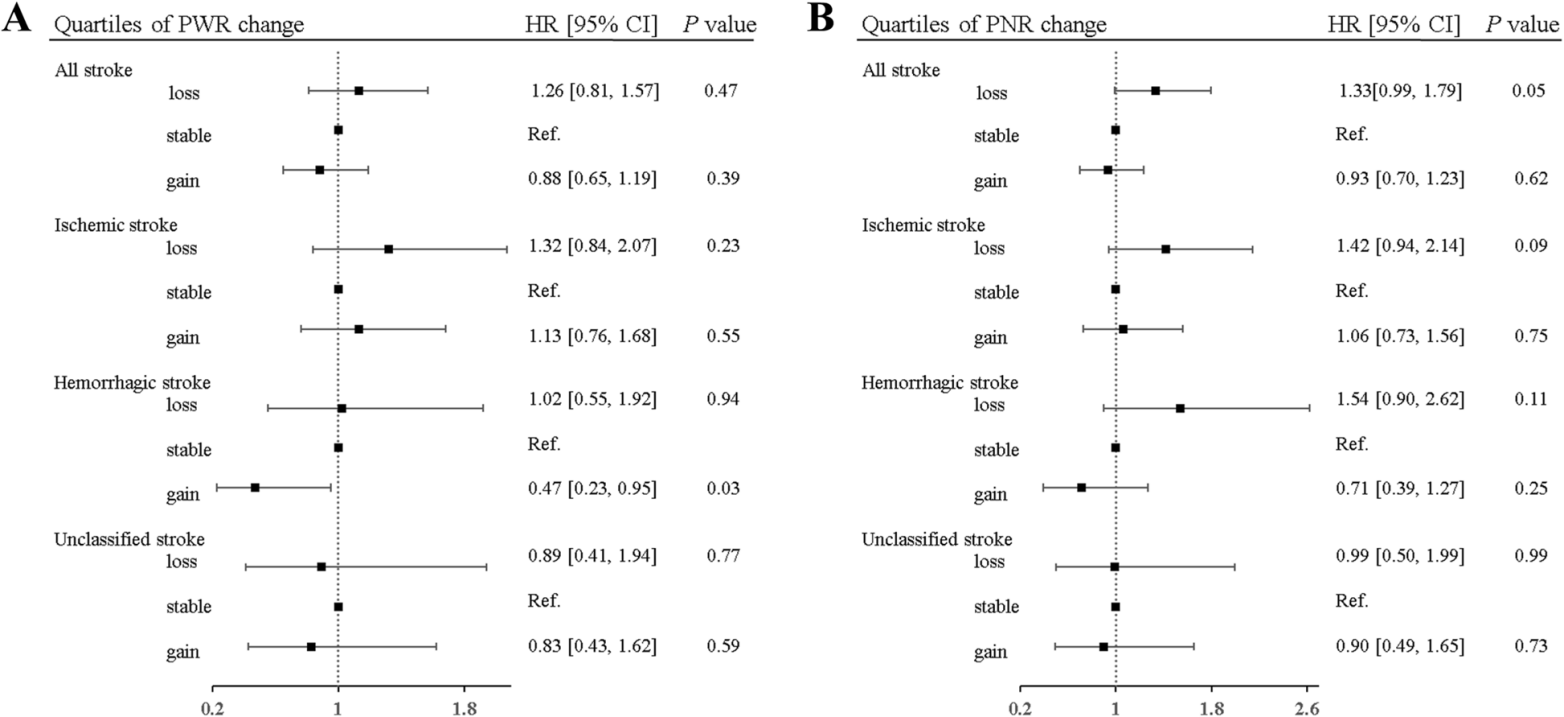
Associations of PWR and PNR changes (-20% to 20%) with fatal stroke among the participants in the first follow-up. This figure plots an adjusted hazard ratio and 95% confidence interval PWR and PNR change alongside with the P value for trend. The adjusted confounders include age, sex, diabetes, hypertension, dyslipidaemia, smoking, alcohol consumption, physical activity, body mass index, self-rated health, cancer, and genitourinary disease(nephropathy, prostatic disease, and gynecologic diseases), chest disease(COPD, chronic bronchitis, emphysema, asthma, tuberculosis, pneumonia), platelet count, and hs-CRP

For dynamic changes, the participants in the 2^nd^, the 3^rd^and the 4^th^ change quartiles of the PWR and the PNR had weak decreasing trends for the risk of fatal all stroke, compared to the participants in the 1^st^ quartile, and significant associations of fatal all stroke risks were observed in those with the highest quartiles for the PWR (aHR = 0.71, 95% CI 0.58–0.93, *P* = 0.03) and the PNR (aHR = 0.73, 95% CI 0.54–1.01, *P* = 0.05) (Table 4). The participants with an increase of 20% for the PWR but a decrease of 20% for the PNR shared respectively the risk of fatal haemarragic stroke (aHR = 0.47, 95% CI 0.22–0.95, *P* = 0.03) and the risk of fatal all stroke (aHR = 1.33, 95% CI 0.99–1.79, *P* = 0.05), compared to the participants with stable levels of their dynamic changes at − 20% ~ 20% (Fig. 3 and Supplementary Table 3).

**Table 4:** Associations of PWR and PNR changes with the risk of fatal stroke occurrence in the GBCS (*n* = 11,038)

	Quartiles of PWR change				Quartiles of PNR change			
	the 1^st^ (≤ -0.11)	the 2^nd^ (−0.11–0.018)	the 3^rd^ (0.018–0.16)	the 4^th^ (≥ 0.16)	the 1^st^ (≤-0.13)	the 2^nd^ (−0.13–0.03)	the 3^rd^ (0.03–0.21)	the 4^th^ (≥0.21)
***All stroke***								
Model 1 (HR, 95% CI)	1.00	0.74 (0.54–1.02), *P* = 0.06	0.75 (0.55–1.03), *P* = 0.07	0.69 (0.50–0.95), *P* = 0.02	1.00	0.77 (0.57–1.05), *P* = 0.09	0.63 (0.46–0.88), *P* = 0.006	0.70 (0.51–0.96), *P* = 0.03
Model 2 (HR, 95% CI)	1.00	0.81 (0.59–1.00), *P* = 0.18	0.85 (0.62–1.16), *P* = 0.29	0.71 (0.51–0.98), *P* = 0.03	1.00	0.86 (0.63–1.17), *P* = 0.34	0.69 (0.50–0.96), *P* = 0.03	0.73 (0.54–1.01), *P* = 0.05
P for trend	0.19				0.10			
***Ischaemic stroke***								
Model 1 (HR, 95% CI)	1.00	0.75 (0.48–1.16), *P* = 0.19	0.72 (0.46–1.12), *P* = 0.14	0.76 (0.49–1.18), *P* = 0.23	1.00	0.67 (0.43–1.06), *P* = 0.08	0.68 (0.44–1.07), *P* = 0.09	0.82 (0.54–1.25), *P* = 0.36
Model 2 (HR, 95% CI)	1.00	0.79 (0.51–1.23), *P* = 0.30	0.81 (0.52–1.26), *P* = 0.34	0.78 (0.50–1.20), *P* = 0.26	1.00	0.74 (0.47–1.17), *P* = 0.20	0.74 (0.47–1.16), *P* = 0.19	0.85 (0.56–1.31), *P* = 0.47
*P* for trend	0.63				0.50			
***Haemorrhagic stroke***								
Model 1 (HR, 95% CI)	1.00	0.66 (0.35–1.21), *P* = 0.17	0.89 (0.51–1.55), *P* = 0.67	0.51 (0.26–0.99), *P* = 0.04	1.00	0.65 (0.36–1.16), *P* = 0.15	0.61 (0.34–1.10), *P* = 0.10	0.44 (0.23–0.85), *P* = 0.01
Model 2 (HR, 95% CI)	1.00	0.71 (0.38–1.31), *P* = 0.27	0.97 (0.55–1.72), *P* = 0.93	0.48 (0.25–0.95), *P* = 0.03	1.00	0.72 (0.40–1.28), *P* = 0.26	0.64 (0.36–1.16), *P* = 0.14	0.44 (0.23–0.85), *P* = 0.01
*P* for trend	0.13				0.09			
***Unclassified stroke***								
Model 1 (HR, 95% CI)	1.00	0.85 (0.44–1.65), *P* = 0.63	0.63 (0.31–1.31), *P* = 0.22	0.75 (0.37–1.49), *P* = 0.41	1.00	1.23 (0.65–2.32), *P* = 0.53	0.52 (0.23–1.17), *P* = 0.11	0.80 (0.39–1.63), *P* = 0.55
Model 2 (HR, 95% CI)	1.00	0.94 (0.48–1.84), *P* = 0.86	0.72 (0.35–1.49), *P* = 0.38	0.77 (0.38–1.55), *P* = 0.50	1.00	1.36 (0.71–2.60), *P* = 0.35	0.57 (0.25–1.29), *P* = 0.18	0.84 (0.41–1.73), *P* = 0.64
*P* for trend	0.79				0.16			

*PWR* platelet-to -white blood cell ratio, *PNR* platelet-to-neutrophil ratio, *hs-CRP* high-sensitivity C-reactive protein, *model 1* a crude hazard ratio model without adjustments, *model 2* a multivariate model adjusted for age, sex, diabetes, hypertension, dyslipidaemia, smoking, alcohol consumption, physical activity, body mass index, self-rated health, cancer, genitourinary disease(nephropathy, prostatic disease, and gynecologic diseases) and chest disease(COPD, chronic bronchitis, emphysema, asthma, tuberculosis, and pneumonia), platelet count, and hs-CRP

## Discussion

We are the first addressing the PWR and the PNR in relation to the risk of fatal stroke occurrence in middle-aged to older populations. In this study, we showed that higher level of the PNR but not the PWR was associated with a decreased risk of fatal all stroke, although the NEUT and the WBC showed a reversed association; and these associations are independent of a series of factors including age, sex, education, occupation, hypertension, diabetes, dyslipidaemia, smoking habit, alcohol consumption, physical activity, BMI, self-rated health, cancer, genitourinary disease, chest disease, platelets and hs-CRP.

In ischemic stroke, the clots were generated to block cerebral arteries including atherosclerosis of great arteries, cardiogenic embolism and small artery occlusion [26] in which atherosclerosis had accompanied with a chronic vascular inflammation or endothelial dysfunction [27]. Lymphocytes and NEUTs took part in the pathogenesis of atherosclerosis [9], and promoted thrombosis formation in stroke and a cardiogenic thromboembolic stroke [28]. In the other hand, the PLTs interacted with a host of leukocytes in thrombocytopenic tissue haemorrhage [29], and the PLT hemITAM (hemi-immunoreceptor tyrosine-based activation motif) signaling took part in vascular barrier integrity [30–33]. Thus, the interaction between LTs and the NEUTs are closely related to stroke in which a chronic inflammation has been in chaperonage.

The WBC and the NEUT have been linked to the risk of stroke events [11, 12, 34–36], and the PLT was closely linked to mortality risks of thrombotic and hemorrhage diseases [16]. We tried firstly to explore the associations of fatal stroke occurrence with the PWR and the PNR who are respectively combined by the PLT and the WBC and the NEUT, and corresponding results should mainly reflect the roles of the WBC and the NEUT again in fatal strokes [25] because we observed significant associations of the WBC and the NEUT but not the PLT with the risks of fatal strokes in relatively healthy middle-aged to older populations. In this study, all of the WBC, the NEUT, and the PNR were related to the risk of fatal stroke occurrence, regardless of a restricted cubic spline model or a quartile model in our study; The PWR and the PNR presented the reversed associations to those of WBC and NEUT who showed similar associations with fatal all stroke and increasing trends in fatal ischaemic stroke. Such results suggest an equal linkage of stroke occurrence to a pre-existing chronic low-grade systemic inflammation in a large cities’ middle-aged to older population. The reasons are that we conducted a further hs-CRP adjustment to exclude acute inflammations, and we used a series of data from relatively healthy elders who had been made an appointment in advance to ensure good health and were able to come the designated place, and the WBC and the NEUT are taken as the denominators in ratios of the PWR and the PNR.

We conducted a large, prospective design for a study of the general Southern Chinese population, and the acquired information allows for systemic adjustments for additional potential confounders in this study because a physical examination and a questionnaire involving a total of 800 questions were completed for all participants. Nevertheless there are limitations in this study. First, we obtained only the death information via record linkage with the GZCDC, and corresponding results, with death as the only outcome, are obviously weakened due to the lack of other outcomes of stroke events. Second, the inaccurate risk factors such as self-rated health may take influences on our results due to a linkage to the objective indicators predicting health status, in addition to a series of potential confounders. Third, the subjects of this study could not represent Chinese individuals due to a limitation of the general populations in South China. Fourth, the unclassified strokes of this study limited the strength to address fatal strokes, especially ischaemic stroke and haemorrhagic stroke.

## Conclusions

Our findings indicated that higher neutrophil count and platelet-to-neutrophil ratio were associated with contrary risks of fatal stroke occurrence, with an increased for the former and a decreased for the later. An asymptomatic chronic low-grade systemic inflammation should therefore play a key role in stroke among relatively healthy middle-aged to older populations.

## Supplementary Information

**Additional file 1:** **Supplementary Table 1.**The 1^st^ follow-up characteristics according to the PWR and PNR changes of participants in the GBCS (*n*=11,038). **Supplementary Table 2. **Associations of WBC, NEUT and PLT with the risk of fatal stroke in the GBCS, 2003-2021 (*n*=27,796). **Supplementary Table 3.** Association of PWR and PNR changes with the risk of fatal stroke occurrence in the GBCS (*n*=11,038).

## Data Availability

The datasets used during the current study are available from the corresponding author on reasonable request.

## Abbreviations

WBC: White blood cell
PLT: Platelet
NEUT: Neutrophil
PWR: Platelet-to-white blood cell ratio
PNR: Platelet-to-neutrophil ratio
hs-CRP: High sensitivity C-reactive protein
ICD: International Classification of Diseases
Ahr: Adjusted hazard ratio
CI: Confidence interval
CVD: Cardiovascular diseases
GBCS: Guangzhou Biobank Cohort Study
GZCDC: Guangzhou Centers for Disease Control and Prevention

## References

[1] Zhou M, Wang H, Zhu J, Chen W, Wang L, Liu S, Li Y, Wang L, Liu Y, Yin P (2016). Cause-specific mortality for 240 causes in China during 1990–2013: a systematic subnational analysis for the Global Burden of Disease Study 2013. Lancet.

[2] Lewington S, Clarke R, Qizilbash N, Peto R, Collins R, Prospective Studies C (2002). Age-specific relevance of usual blood pressure to vascular mortality: a meta-analysis of individual data for one million adults in 61 prospective studies. Lancet.

[3] Bragg F, Holmes MV, Iona A, Guo Y, Du H, Chen Y, Bian Z, Yang L, Herrington W, Bennett D (2017). Association Between Diabetes and Cause-Specific Mortality in Rural and Urban Areas of China. JAMA.

[4] Koton S, Schneider AL, Rosamond WD, Shahar E, Sang Y, Gottesman RF, Coresh J (2014). Stroke incidence and mortality trends in US communities, 1987 to 2011. JAMA.

[5] Chen Z, Peto R, Zhou M, Iona A, Smith M, Yang L, Guo Y, Chen Y, Bian Z, Lancaster G (2015). Contrasting male and female trends in tobacco-attributed mortality in China: evidence from successive nationwide prospective cohort studies. Lancet.

[6] Thun MJ, Apicella LF, Henley SJ (2000). Smoking vs other risk factors as the cause of smoking-attributable deaths: confounding in the courtroom. JAMA.

[7] Portegies ML, Bos MJ, Hofman A, Heeringa J, Franco OH, Koudstaal PJ, Ikram MA (2016). Role of Prestroke Vascular Pathology in Long-Term Prognosis After Stroke: The Rotterdam Study. Stroke.

[8] Vaccarezza M, Balla C, Rizzo P (2018). Atherosclerosis as an inflammatory disease: Doubts? No more. International journal of cardiology Heart & vasculature.

[9] Ross R (1999). Atherosclerosis–an inflammatory disease. N Engl J Med.

[10] Libby P, Ridker PM, Hansson GK (2011). Progress and challenges in translating the biology of atherosclerosis. Nature.

[11] Brown DW, Ford ES, Giles WH, Croft JB, Balluz LS, Mokdad AH (2004). Associations between white blood cell count and risk for cerebrovascular disease mortality: NHANES II Mortality Study, 1976–1992. Ann Epidemiol.

[12] Jee SH, Park JY, Kim HS, Lee TY, Samet JM (2005). White blood cell count and risk for all-cause, cardiovascular, and cancer mortality in a cohort of Koreans. Am J Epidemiol.

[13] Doring Y, Soehnlein O, Weber C (2017). Neutrophil Extracellular Traps in Atherosclerosis and Atherothrombosis. Circ Res.

[14] Quillard T, Franck G, Mawson T, Folco E, Libby P (2017). Mechanisms of erosion of atherosclerotic plaques. Curr Opin Lipidol.

[15] Fuentes QE, Fuentes QF, Andres V, Pello OM (2013). Font de Mora J, Palomo GI: Role of platelets as mediators that link inflammation and thrombosis in atherosclerosis. Platelets.

[16] Tsai MT, Chen YT, Lin CH, Huang TP, Tarng DC (2015). Taiwan Geriatric Kidney Disease Research G: U-shaped mortality curve associated with platelet count among older people: a community-based cohort study. Blood.

[17] Fang HY, Lin CY, Ko WJ (2005). Hematology and coagulation parameters predict outcome in Taiwanese patients with spontaneous intracerebral hemorrhage. Eur J Neurol.

[18] Jie Y, Gong J, Xiao C, Zhu S, Zhou W, Luo J, Chong Y, Hu B (2018). Low Platelet to White Blood Cell Ratio Indicates Poor Prognosis for Acute-On-Chronic Liver Failure. Biomed Res Int.

[19] Schulz GB, Grimm T, Buchner A, Jokisch F, Grabbert M, Schneevoigt BS, Kretschmer A, Stief CG, Karl A (2017). Prognostic Value of the Preoperative Platelet-to-leukocyte Ratio for Oncologic Outcomes in Patients Undergoing Radical Cystectomy for Bladder Cancer. Clin Genitourin Cancer.

[20] Chen Z, Huang Y, Li S, Lin J, Liu W, Ding Z, Li X, Chen Y, Pang W, Yang D (2016). Platelet-to-White Blood Cell Ratio: A Prognostic Predictor for 90-Day Outcomes in Ischemic Stroke Patients with Intravenous Thrombolysis. Journal of stroke and cerebrovascular diseases : the official journal of National Stroke Association.

[21] Wei XB, Liu YH, He PC, Yu DQ, Tan N, Zhou YL, Chen JY (2017). The impact of admission neutrophil-to-platelet ratio on in-hospital and long-term mortality in patients with infective endocarditis. Clin Chem Lab Med.

[22] Long H, Qin K, Chen J, Chen Y, Chen L, Zeng J, Liang Z (2018). Biomarkers of gastric cancer-related ischemic stroke and its underlying pathogenesis. Medicine.

[23] Jiang C, Thomas GN, Lam TH, Schooling CM, Zhang W, Lao X, Adab P, Liu B, Leung GM, Cheng KK (2006). Cohort profile: The Guangzhou Biobank Cohort Study, a Guangzhou-Hong Kong-Birmingham collaboration. Int J Epidemiol.

[24] Deng HB, Macfarlane DJ, Thomas GN, Lao XQ, Jiang CQ, Cheng KK, Lam TH (2008). Reliability and validity of the IPAQ-Chinese: the Guangzhou Biobank Cohort study. Med Sci Sports Exerc.

[25] Hu ZB, Lu ZX, Zhu F, Jiang CQ, Zhang WS, Pan J, Jin YL, Xu L, Thomas GN, Cheng K (2021). Higher total white blood cell and neutrophil counts are associated with an increased risk of fatal stroke occurrence: the Guangzhou biobank cohort study. BMC Neurol.

[26] Adams HP, Bendixen BH, Kappelle LJ, Biller J, Love BB, Gordon DL, Marsh EE (1993). 3rd: Classification of subtype of acute ischemic stroke. Definitions for use in a multicenter clinical trial. TOAST. Trial of Org 10172 in Acute Stroke Treatment. Stroke.

[27] Gistera A, Hansson GK (2017). The immunology of atherosclerosis. Nat Rev Nephrol.

[28] Laridan E, Denorme F, Desender L, Francois O, Andersson T, Deckmyn H, Vanhoorelbeke K, De Meyer SF (2017). Neutrophil extracellular traps in ischemic stroke thrombi. Ann Neurol.

[29] Hillgruber C, Poppelmann B, Weishaupt C, Steingraber AK, Wessel F, Berdel WE, Gessner JE, Ho-Tin-Noe B, Vestweber D, Goerge T (2015). Blocking neutrophil diapedesis prevents hemorrhage during thrombocytopenia. J Exp Med.

[30] Rahman M, Zhang S, Chew M, Ersson A, Jeppsson B, Thorlacius H (2009). Platelet-derived CD40L (CD154) mediates neutrophil upregulation of Mac-1 and recruitment in septic lung injury. Ann Surg.

[31] Duerschmied D, Suidan GL, Demers M, Herr N, Carbo C, Brill A, Cifuni SM, Mauler M, Cicko S, Bader M (2013). Platelet serotonin promotes the recruitment of neutrophils to sites of acute inflammation in mice. Blood.

[32] Perazzio SF, Soeiro-Pereira PV, Dos Santos VC, de Brito MV, Salu B, Oliva MLV, Stevens AM, de Souza AWS, Ochs HD, Torgerson TR (2017). Soluble CD40L is associated with increased oxidative burst and neutrophil extracellular trap release in Behcet's disease. Arthritis Res Ther.

[33] Yokoyama S, Ikeda H, Haramaki N, Yasukawa H, Murohara T, Imaizumi T (2005). Platelet P-selectin plays an important role in arterial thrombogenesis by forming large stable platelet-leukocyte aggregates. J Am Coll Cardiol.

[34] Qu X, Shi J, Cao Y, Zhang M, Xu J (2018). Prognostic Value of White Blood Cell Counts and C-reactive Protein in Acute Ischemic Stroke Patients After Intravenous Thrombolysis. Curr Neurovasc Res.

[35] Fang YN, Tong MS, Sung PH, Chen YL, Chen CH, Tsai NW, Huang CJ, Chang YT, Chen SF, Chang WN (2017). Higher neutrophil counts and neutrophil-to-lymphocyte ratio predict prognostic outcomes in patients after non-atrial fibrillation-caused ischemic stroke. Biomed J.

[36] Furlan JC, Vergouwen MD, Fang J, Silver FL (2014). White blood cell count is an independent predictor of outcomes after acute ischaemic stroke. Eur J Neurol.

