# Supplementary material for: Association of platelet-to-white blood cell ratio and platelet-to-neutrophil ratio with the risk of fatal stroke occurrence in middle-aged to older Chinese
Source: BMC Geriatr. 2022 May 17;22:430. doi: 10.1186/s12877-022-03134-z (PMC9112464; doi:10.1186/s12877-022-03134-z)
Supplement: Supplementary file 1 — Additional file 1: Supplementary Table 1.The 1st follow-up characteristics according to the PWR and PNR changes of participants in the GBCS (n=11,038). Supplementary Table 2. Associations of WBC, NEUT and PLT with the risk of fatal stroke in the GBCS, 2003-2021 (n=27,796). Supplementary Table 3. Association of PWR and PNR changes with the risk of fatal stroke occurrence in the GBCS (n=11,038). [file 12877_2022_3134_MOESM1_ESM.docx]

**Supplementary Table 1** The 1^st^ follow-up characteristics according to the PWR and PNR changes of participants in the GBCS (n=11,038)

| Characteristics | Loss  (<−20%) | Stable  (−20%–20%) | Gain  (>20%) | *_P_* _value_ | the 1^st^  (≤−0.11) | the 2^nd^  (−0.11–0.01) | the 3^rd^  (0.01–0.16) | the 4^th^  (≥0.16) | *P* value |
| --- | --- | --- | --- | --- | --- | --- | --- | --- | --- |
| ***the PWR*** | | | | | | | | | |
| Number, n | 1341 | 7470 | 2227 |  | 2759 | 2760 | 2758 | 2761 |  |
| Age (years) | 65.7±6.9 | 65.7±6.8 | 65.9±6.8 | <0.001 | 66.3±6.9 | 65.7±6.9 | 65.5±6.7 | 65.9±6.8 | <0.001 |
| Sex, male (%) | 357 (26.6) | 2013 (26.9) | 681 (30.6) | 0.002 | 756 (27.4) | 728 (26.4) | 727 (26.4) | 840 (30.4) | 0.002 |
| Smoking, n (%) |  |  |  | <0.001 |  |  |  |  | <0.001 |
| Never | 1046 (78.0) | 5923 (79.3) | 1647 (74.0) |  | 2185 (79.2) | 2186 (79.2) | 2184 (79.2) | 2061 (74.6) |  |
| Former | 121 (9.0) | 582 (7.8) | 201 (9.0) |  | 224 (8.1) | 227 (8.2) | 206 (7.5) | 247 (8.9) |  |
| Current | 174 (13.0) | 965 (12.9) | 379 (17.0) |  | 350 (12.7) | 347 (12.6) | 368 (13.3) | 453 (16.4) |  |
| Alcohol drinking, n (%) |  |  |  | 0.09 |  |  |  |  | 0.72 |
| Never | 764 (57.0) | 4227 (56.6) | 1295 (58.1) |  | 1584 (57.4) | 1568 (56.8) | 1547 (56.1) | 1587 (57.5) |  |
| Former | 385 (28.7) | 2319 (31.0) | 646 (29.0) |  | 811 (29.4) | 856 (31.0) | 857 (31.1) | 826 (29.9) |  |
| Current | 192 (14.3) | 924 (12.4) | 286 (12.8) |  | 364 (13.2) | 336 (12.2) | 354 (12.8) | 348 (12.6) |  |
| Physical activity,*IPAQ*, n (%) |  |  |  | 0.72 |  |  |  |  | 0.26 |
| Inactive | 41 (3.1) | 206 (2.8) | 58 (2.6) |  | 79 (2.9) | 77 (2.8) | 77 (2.8) | 72 (2.6) |  |
| Moderate active | 323 (24.1) | 1726 (23.1) | 538 (24.2) |  | 690 (25.0) | 617 (22.4) | 621 (22.5) | 659 (23.9) |  |
| Active | 977 (72.9) | 5538 (74.1) | 1631 (73.2) |  | 1990 (72.1) | 2066 (74.9) | 2060 (74.7) | 2030 (73.5) |  |
| Body mass index, kg/㎡ |  |  |  | <0.001 |  |  |  |  | 0.03 |
| <18.5 | 82 (6.1) | 420 (5.6) | 153 (6.9) |  | 150 (5.4) | 164 (5.9) | 157 (5.7) | 184 (6.7) |  |
| 18.5-23.9 | 657 (49.0) | 3547 (47.5) | 1148 (51.5) |  | 1313 (47.6) | 1345 (48.7) | 1299 (47.1) | 1395 (50.5) |  |
| 24-27.9 | 447 (33.3) | 2712 (36.3) | 727 (32.6) |  | 998 (36.2) | 977 (35.4) | 984 (35.7) | 927 (33.6) |  |
| ≥28 | 155 (11.6) | 791 (10.6) | 199 (8.9) |  | 298 (10.8) | 274 (9.9) | 318 (11.5) | 255 (9.2) |  |
| Self-rated health, n (% )(good/very good) | 1168 (87.1) | 6580 (88.1) | 1928 (86.6) | 0.13 | 2409 (87.3) | 2444 (88.6) | 2425 (87.9) | 2398 (86.9) | 0.24 |
| Hypertension, n (%) | 688 (51.3) | 3771 (50.5) | 1090 (48.9) | 0.32 | 1415 (51.3) | 1363 (49.4) | 1401 (50.8) | 1370 (49.6) | 0.43 |
| Diabetes, n (% ) | 228 (17.0) | 1207 (16.2) | 391 (17.6) | 0.26 | 430 (15.6) | 474 (17.2) | 429 (15.6) | 493 (17.9) | 0.04 |
| Dyslipidemia, n (%) | 1213 (90.5) | 6893 (92.3) | 2033 (91.3) | 0.04 | 2521 (91.4) | 2565 (92.9) | 2535 (91.9) | 2518(91.2) | 0.08 |
| Cancer, n (% ) | 39 (2.9) | 205 (2.7) | 60 (2.7) | 0.93 | 83 (3.0) | 82 (3.0) | 65 (2.4) | 74 (2.7) | 0.42 |
| GD, n (% ) | 458 (34.2) | 2716 (36.4) | 816 (36.6) | 0.26 | 1017 (36.9) | 1000 (36.2) | 955 (34.6) | 1018 (36.9) | 0.26 |
| Chest disease, n (% ) | 336 (25.1) | 1675 (22.4) | 515 (23.1) | 0.10 | 670 (24.3) | 602 (21.8) | 622 (22.6) | 632 (22.9) | 0.17 |
| WBC, *10^9/L | 7.1±2.0 | 6.3±1.4 | 5.9±1.5 | <0.001 | 6.8±1.8 | 6.3±1.4 | 6.2±1.3 | 5.9±1.4 | <0.001 |
| NEUT, *10^9/L | 4.4±1.7 | 3.7±1.1 | 3.5±1.1 | <0.001 | 4.1±1.5 | 3.8±1.1 | 3.6±1.0 | 3.5±1.1 | <0.001 |
| PLT, *10^9/L | 206.2±59.4 | 229.9±54.8 | 246.0±73.1 | <0.001 | 214.4±56.6 | 225.1±50.2 | 236.7±58.2 | 245.1±70.7 | <0.001 |
| All stroke, n (%) | 44 (3.3) | 195 (2.6) | 53 (2.4) | 0.25 | 92 (3.3) | 68 (2.5) | 69 (2.5) | 63 (2.3) | 0.07 |
| Ischaemic stroke, n (%) | 24 (1.8) | 95 (1.3) | 33 (1.5) | 0.29 | 47 (1.7) | 35 (1.3) | 34 (1.2) | 36 (1.3) | 0.38 |
| Haemorrhagic stroke, n (%) | 12 (0.9) | 58 (0.8) | 9 (0.4) | 0.13 | 26 (1.0) | 17 (0.6) | 23 (0.8) | 13 (0.5) | 0.15 |
| Unclassified stroke, n (%) | 8 (0.6) | 42 (0.6) | 11 (0.5) | 0.90 | 19 (0.7) | 16 (0.6) | 12 (0.4) | 14 (0.5) | 0.61 |
| ***the PNR*** | | | | | | | | | |
| Number, n | 1812 | 6346 | 2880 |  | 2758 | 2761 | 2759 | 2760 |  |
| Age (years) | 65.8±6.8 | 66.3±6.9 | 65.9±6.8 | 0.008 | 66.3±6.8 | 65.6±6.8 | 65.8±6.8 | 65.8±6.9 | 0.003 |
| Sex, male (%) | 466 (25.7) | 1744 (27.5) | 841 (29.2) | 0.03 | 728 (26.4) | 758 (27.5) | 762 (27.6) | 803 (29.1) | 0.16 |
| Smoking, n (%) |  |  |  | 0.01 |  |  |  |  | 0.08 |
| Never | 1444 (79.7) | 4980 (78.5) | 2192 (76.1) |  | 2189 (79.4) | 2168 (78.5) | 2153 (78.0) | 2106 (76.3) |  |
| Former | 144 (7.9) | 520 (8.2) | 240 (8.3) |  | 229 (8.2) | 229 (8.3) | 220 (8.0) | 230 (8.3) |  |
| Current | 224 (12.4) | 846 (13.3) | 448 (15.6) |  | 344 (12.5) | 364 (13.2) | 386 (14.0) | 424 (15.4) |  |
| Alcohol drinking, n (%) |  |  |  | 0.93 |  |  |  |  | 0.94 |
| Never | 1039 (57.3) | 3591 (56.6) | 1656 (57.5) |  | 1570 (56.9) | 1570 (56.9) | 1570 (56.9) | 1576 (57.1) |  |
| Former | 545 (30.1) | 1944 (30.6) | 861 (29.9) |  | 854 (31.0) | 832 (30.1) | 829 (30.0) | 835 (30.3) |  |
| Current | 228 (12.6) | 811 (12.8) | 363 (12.6) |  | 334 (12.1) | 359 (13.0) | 360 (13.0) | 349 (12.6) |  |
| Physical activity,*IPAQ*, n (%) |  |  |  | 0.50 |  |  |  |  | 0.29 |
| Inactive | 61 (3.4) | 167 (2.6) | 77 (2.7) |  | 82 (3.0) | 65 (2.4) | 87 (3.2) | 71 (2.6) |  |
| Moderate active | 426 (23.5) | 1476 (23.3) | 685 (23.8) |  | 665 (24.1) | 617 (22.3) | 649 (23.5) | 656 (23.8) |  |
| Active | 1325 (73.1) | 4703 (74.1) | 2118 (73.5) |  | 2011 (72.9) | 2079 (75.3) | 2023 (73.3) | 2033 (73.7) |  |
| Body mass index, kg/㎡ |  |  |  | 0.04 |  |  |  |  | 0.01 |
| <18.5 | 111 (6.1) | 356 (5.6) | 188 (6.5) |  | 166 (6.0) | 1052 (5.5) | 152 (5.5) | 185 (6.7) |  |
| 18.5-23.9 | 871 (48.1) | 3030 (47.7) | 1451 (50.4) |  | 1333 (48.3) | 1288 (46.6) | 1335 (48.4) | 1396 (50.6) |  |
| 24-27.9 | 634 (35.0) | 2279 (34.9) | 973 (33.8) |  | 973 (35.3) | 1000 (36.2) | 993 (36.0) | 920 (33.3) |  |
| ≥28 | 196 (10.8) | 681 (10.7) | 268 (9.3) |  | 286 (10.4) | 321 (11.6) | 279 (10.1) | 259 (9.4) |  |
| Self-rated health, n (% )(good/very good) | 1577 (87.0) | 5570 (87.8) | 2529 (87.8) | 0.67 | 2394 (86.8) | 2433 (88.1) | 2433 (88.2) | 2416 (87.5) | 0.37 |
| Hypertension, n (%) | 938 (51.8) | 3207 (50.5) | 1404 (48.8) | 0.11 | 1443 (52.3) | 1376 (49.8) | 1392 (50.5) | 1338 (48.5) | 0.03 |
| Diabetes, n (% ) | 315 (17.4) | 1019 (16.1) | 492 (17.1) | 0.27 | 469 (17.0) | 444 (16.1) | 447 (16.2) | 466 (16.9) | 0.70 |
| Dyslipidemia, n (%) | 1651 (91.1) | 5857 (92.3) | 2631 (91.4) | 0.14 | 2534 (91.9) | 2539 (92.0) | 2542 (92.1) | 2524 (91.4) | 0.82 |
| Cancer, n (% ) | 55 (3.0) | 182 (2.9) | 67 (2.3) | 0.24 | 85 (3.1) | 76 (2.8) | 77 (2.8) | 66 (2.4) | 0.48 |
| GD, n (% ) | 665 (36.7) | 2292 (36.1) | 1033 (35.9) | 0.84 | 1001 (36.3) | 1008 (36.5) | 998 (36.2) | 983 (35.6) | 0.91 |
| Chest disease, n (% ) | 440 (24.3) | 1447 (22.8) | 639 (22.2) | 0.24 | 651 (23.6) | 635 (23.0) | 623 (22.6) | 617 (22.4) | 0.70 |
| WBC, *10^9/L | 7.0±1.9 | 6.3±1.4 | 5.9±1.4 | <0.001 | 6.8±1.8 | 6.4±1.4 | 6.2±1.3 | 5.9±1.4 | <0.001 |
| NEUT, *10^9/L | 4.4±1.7 | 3.8±1.0 | 3.4±1.1 | <0.001 | 4.3±1.5 | 3.8±1.1 | 3.6±1.0 | 3.3±1.1 | <0.001 |
| PLT, *10^9/L | 215.6±59.8 | 230.5±57.0 | 239.1±66.5 | <0.001 | 218.9±57.8 | 227.2±51.7 | 235.9±62.4 | 239.2±67.0 | <0.001 |
| All stroke, n (%) | 64 (3.5) | 158 (2.5) | 70 (2.4) | 0.03 | 93 (3.4) | 72 (2.6) | 60 (2.2) | 67 (2.4) | 0.03 |
| Ischaemic stroke, n (%) | 33 (1.9) | 79 (1.3) | 40 (1.4) | 0.17 | 47 (1.7) | 32 (1.2) | 33 (1.2) | 40 (1.5) | 0.26 |
| Haemorrhagic stroke, n (%) | 20 (1.1) | 44 (0.7) | 15 (0.5) | 0.06 | 29 (1.1) | 19 (0.7) | 18 (0.7) | 13 (0.5) | 0.07 |
| Unclassified stroke, n (%) | 11 (0.6) | 35 (0.6) | 15 (0.5) | 0.91 | 17 (0.6) | 21 (0.8) | 9 (0.3) | 14 (0.5) | 0.16 |

Hypertension: systolic blood pressure ≥140 mmHg, diastolic blood pressure ≤90 mmHg, medication or diagnosis; diabetes: fasting blood glucose ≥7, medication or diagnosis; dyslipidaemia: total cholesterol ≥5.2 mmol/L, triglyceride ≥1.7 mmol/L, low density lipoprotein ≥3.4 mmol/L, high density lipoprotein <1.0 mmol/L, medication or diagnosis; NUET: neutrophil; WBC: white blood cell; CRP: C-reactive protein; GD: genitourinary disease (including nephropathy, prostatic disease, and gynaecologic diseases); chest disease (including chronic obstructive pulmonary disease, chronic bronchitis, emphysema, asthma, tuberculosis, and pneumonia).

**Supplementary Table 2** Associations of WBC, NEUT and PLT with the risk of fatal stroke in the GBCS, 2003-2021 (n=27,796)

|  | | Quartiles of WBC (*10^9/L) | | | | | | | | | | *P* value trend | | | Quartiles of NEUT (*10^9/L) | | | | | | | | | | *P* value trend | | |  | | Quartiles of PLT (*10^9/L) | | | | | | | | | *P* value trend | | |
| --- | --- | --- | --- | --- | --- | --- | --- | --- | --- | --- | --- | --- | --- | --- | --- | --- | --- | --- | --- | --- | --- | --- | --- | --- | --- | --- | --- | --- | --- | --- | --- | --- | --- | --- | --- | --- | --- | --- | --- | --- | --- |
|  |  | the 1^st^ (<5.3) | | | | the  2^nd^(5.3-6.1) | | the 3^rd^ (6.2-7.2) | | the 4^th^(>7.2) | |  |  |  | the 1^st^(<3.0) | | | | the 2^nd^ (3.0-3.6) | | the 3^rd^ (3.7-4.4) | | the 4^th^ (>4.5) | |  |  |  | the 1^st^  (<190) | | | | the 2^nd^  (191-223) | | the 3^rd^  (224-260) | | | the 4^th^ (>260) | |  |  |  |
| ***All stroke*** | | | | | | | | | | | | | | | | | | | | | | | | | | | | | | | | | | | | | | | | | |
| Person years | | 100,332 | | | | 111,351 | | 91,360 | | 96,072 | | |  | |  | | 106,915 | | 89,125 | | 108,359 | | 94,718 | | |  | |  | | 100,941 | | 99,855 | | | 100,652 | | 97,668 | |  | | |
| per 10^5 person-years | | | 164.4 | | | 167.9 | | 211.2 | | 304.9 | | |  | |  | | 143.1 | | 186.2 | | 200.3 | | 318.8 | | |  | |  | | 244.7 | | 189.3 | | | 205.6 | | 199.6 | |  | | |
| No. of deaths | | 165 | | | | 187 | | 193 | | 293 | | |  | |  | | 153 | | 166 | | 217 | | 302 | | |  | |  | | 247 | | 189 | | | 207 | | 195 | |  | | |
| Model 1  (HR; 95% CI) | | Ref. | | | | 1.01  (0.82-1.25) | | 1.28  (1.04-1.58)^a^ | | 1.86  (1.54-2.26)^c^ | | | < 0.001 | | | | Ref. | | 1.30  (1.04-1.62^a^ | | 1.40 (1.13-1.72)^b^ | | 2.24 (1.85-2.73)^c^ | | | < 0.001 | | | | Ref. | | 0.77(0.64-0.93)^b^ | | | 0.84 (0.70-1.01) | | 0.82 (0.68-0.98)^a^ | | 0.03 | | |
| *P* value | |  | | | | 0.89 | | 0.02 | | < 0.001 | | |  | |  | |  | | 0.02 | | 0.002 | | < 0.001 | | |  | |  | |  | | 0.007 | | | 0.06 | | 0.03 | |  | | |
| Model 2  (HR; 95% CI) | | Ref. | | | | 0.89  (0.72-1.10) | | 1.04  (0.84-1.29) | | 1.35  (1.09-1.66)^b^ | | | < 0.001 | | | | Ref. | | 1.09  (0.88-1.37) | | 1.08 (0.87-1.34) | | 1.45 (1.18-1.79)^c^ | | | 0.001 | |  | | Ref. | | 0.93 (0.77-1.13) | | | 1.07 (0.88-1.29) | | 1.17 (0.97-1.43) | | 0.13 | | |
| *P* value | |  | | | | 0.28 | | 0.71 | | 0.005 | | |  | |  | |  | | 0.80 | | 0.68 | | <0.001 | | |  | |  | | . | | 0.45 | | | 0.50 | | 0.10 | |  | | |
| ***Ischaemic stroke*** | | | | | | | | | | | | | | | | | | | | | | | | | | | | | | | | | | | | | | | | | |
| Person years | | 99,489 | | | | 110,242 | | 90,482 | | 94,691 | | |  | |  | | 106,105 | | 88,296 | | 107,110 | | 93,394 | | |  | |  | | 99,742 | | 98,839 | | | 99,656 | | 96,668 | |  | | |
| per 10^5 person-years | | | 84.4 | | | 72.6 | | 110.5 | | 157.3 | | |  | |  | | 67.8 | | 95.1 | | 88.7 | | 173.4 | | |  | |  | | 123.1 | | 91.0 | | | 110.3 | | 93.1 | |  | | |
| No. of deaths | | 84 | | | | 80 | | 100 | | 149 | | |  | |  | | 72 | | 84 | | 95 | | 162 | | |  | |  | | 123 | | 90 | | | 110 | | 90 | |  | | |
| Model 1  (HR; 95% CI) | | Ref. | | | | 0.85  (0.63-1.16) | | 1.30  (0.97-1.74) | | 1.87  (1.43-2.44)^c^ | | | < 0.001 | | | | Ref. | | 1.40  (1.02-1.92)^a^ | | 1.30 (0.96-1.77) | | 2.57 (1.95-3.39)^c^ | | | < 0.001 | | | | Ref. | | 0.73 (0.56-0.96)^a^ | | | 0.89 (0.69-1.16) | | 0.76 (0.58-0.99)^a^ | | 0.09 | | |
| *P* value | |  | | | | 0.30 | | 0.07 | | < 0.001 | | |  | |  | |  | | 0.03 | | 0.09 | | < 0.001 | | |  | |  | |  | | 0.03 | | | 0.40 | | 0.04 | |  | | |
| Model 2  (HR; 95% CI) | | Ref. | | | | 0.73  (0.53-0.99)^a^ | | 1.02  (0.75-1.37) | | 1.28  (0.96-1.71) | | | 0.002 | |  | | Ref. | | 1.15  (0.83-1.58) | | 0.97 (0.71-1.33) | | 1.58 (1.17-2.12)^a^ | | | 0.001 | |  | | Ref. | | 0.89 (0.68-1.17) | | | 1.15 (0.88-1.49) | | 1.12 (0.85-1.48) | | 0.28 | | |
| *P* value | |  | | | | 0.04 | | 0.91 | | 0.09 | | |  | |  | |  | | 0.39 | | 0.87 | | 0.03 | | |  | |  | |  | | 0.42 | | | 0.29 | | 0.43 | |  | | |
| ***Haemorrhagic stroke*** | | | | | | | | | | | | | | | | | | | | | | | | | | | | | | | | | | | | | | | | | |
| Person years | | 99,131 | | | | 110,022 | | 89,802 | | 93,860 | | |  | |  | | 105,883 | | 87,825 | | 106,745 | | 92,362 | | |  | |  | | 99,209 | | 98,416 | | | 98,967 | | 96,223 | |  | | |
| per 10^5 person-years | | | | 57.5 | | 58.2 | | 60.1 | | 94.8 | | |  | |  | | 57.6 | | 53.5 | | 65.6 | | 93.1 | | |  | |  | | 83.7 | | 64.0 | | | 59.6 | | 61.3 | |  | | |
| No. of deaths | | 57 | | | | 64 | | 54 | | 89 | | |  | |  | | 61 | | 47 | | 70 | | 86 | | |  | |  | | 83 | | 63 | | | 59 | | 59 | |  | | |
| Model 1  (HR; 95% CI) | | Ref. | | | | 1.00  (0.71-1.44) | | 1.04  (0.72-1.52) | | 1.66  (1.19-2.31)^b^ | | | 0.003 | |  | | Ref. | | 0.93  (0.63-1.36) | | 1.40  (0.81-1.61) | | 1.63 (1.17-2.26)^b^ | | | 0.004 | |  | | Ref. | | 0.76 (0.55-1.05) | | | 0.71 (0.51-0.99) | | 0.73  (0.52-1.02)^b^ | | 0.13 | | |
| *P* value | |  | | | | 0.96 | | 0.82 | | 0.003 | | |  | |  | |  | | 0.70 | | 0.46 | | 0.004 | | |  | |  | |  | | 0.10 | | | 0.04 | | 0.06 | |  | | |
| Model 2  (HR; 95% CI) | | Ref. | | | | 0.96  (0.67-1.37) | | 0.97  (0.66-1.42) | | 1.43  (0.99-2.05) | | | 0.053 | |  | | Ref. | | 0.84  (0.57-1.23) | | 0.98 (0.67-1.39) | | 1.23 (0.86-1.75) | | | 0.20 | |  | | Ref. | | 0.91 (0.66-1.27) | | | 0.88 (0.63-1.24) | | 1.00 (0.71-1.41) | | 0.84 | | |
| *P* value | |  | | | | 0.81 | | 0.86 | | 0.06 | | |  | |  | |  | | 0.36 | | 0.89 | | 0.25 | | |  | |  | |  | | 0.59 | | | 0.46 | | 0.98 | |  | | |
| ***Unclassified stroke*** | | | | | | | | | | | | | | | | | | | | | | | | | | | | | | | | | | | | | | | | | |
| Person years | | 98,794 | | | | 109,798 | | 89,758 | | 93,546 | | |  | |  | | 105,499 | | 87,700 | | 106,557 | | 92,140 | | |  | |  | | 98,814 | | 98,208 | | | 98,754 | | 96,120 | |  | | |
| per 10^5 person-years | | 24.3 | | | | 39.2 | | 43.4 | | 58.8 | | |  | |  | | 18.9 | | 39.9 | | 48.8 | | 58.6 | | |  | |  | | 41.5 | | 36.6 | | | 38.5 | | 47.8 | |  | | |
| No. of deaths | | | | 24 | | 43 | | 39 | | 55 | | |  | |  | | 20 | | 35 | | 52 | | 54 | | |  | |  | | 41 | | 36 | | | 38 | | 46 | |  |  |  |
| Model 1  (HR; 95% CI) | | | | Ref. | | 1.60  (0.97-2.64) | | 1.78  (1.07-2.96)^a^ | | 2.42  (1.50-3.91)^c^ | | | 0.003 | |  | | Ref. | | 2.09  (1.21-3.63)^b^ | | 2.56 (1.53-4.29)^c^ | | 3.09 (1.85-5.17)^c^ | | | <0.001 | |  | | Ref. | | 0.88 (0.56-1.37) | | | 0.92 (0.59-1.43) | | 1.15 (0.75-1.75) | | 0.63 |  |  |
| *P* value | | | |  | | 0.06 | | 0.03 | | <0.001 | | |  | |  | |  | | 0.008 | | < 0.001 | | < 0.001 | | |  | |  | |  | | 0.57 | | | 0.72 | | 0.52 | |  |  |  |
| Model 2  (HR; 95% CI) | | | | Ref. | | 1.31  (0.79-2.17) | | 1.33  (0.79-2.24) | | 1.54  (0.93-2.57) | | | 0.42 | |  | | Ref. | | 1.69  (0.97-2.95) | | 1.86  (1.10-3.14)^a^ | | 1.80  (1.05-3.07)^a^ | | | 0.12 | |  | | Ref. | | 1.08 (0.69-1.70) | | | 1.20 (0.77-1.88) | | 1.72 (1.11-2.65)^a^ | | 0.07 |  |  |
| *P* value | | | |  | | 0.29 | | 0.28 | | 0.09 | | |  | |  | |  | | 0.06 | | 0.02 | | 0.03 | | |  | |  | |  | | 0.72 | | | 0.42 | | 0.01 | |  |  |  |

Ref: reference; ^C^ *P*< 0.001,^b^ *P*< 0.01,^a^*P*< 0.05; model 1: a crude hazard ratio model without adjustment for confounders; model 2: a multivariate model adjusted for sex, age, diabetes, hypertension, dyslipidaemia, smoking, alcohol consumption, physical activity, body mass index, self-rated health, cancer, genitourinary disease (including nephropathy, prostatic disease, and gynaecologic diseases), chest disease (including chronic obstructive pulmonary disease, chronic bronchitis, emphysema, asthma, tuberculosis, and pneumonia) and platelet count.

**Supplementary Table 3** Association of PWR and PNR changes with the risk of fatal stroke occurrence in the GBCS (n=11,038)

|  |  | PWR change |  |  |  | PNR change |  |
| --- | --- | --- | --- | --- | --- | --- | --- |
|  | Loss  (<−5%) | Stable  (−5% –5%) | Gain  (>5%) |  | Loss  (<−5%) | Stable  (−5% –5%) | Gain  (>5%) |
| ***All stroke*** | |  |  |  |  |  |  |
| Model 1 (HR, 95% CI) | 1.30 (0.94–1.81), *P*=0.11 | Ref. | 1.04 (0.74–1.44), *P*= 0.84 |  | 1.54 (1.07–2.21), *P*=0.02 | Ref. | 1.12 (0.78–1.62), *P*= 0.53 |
| Model 3 (HR, 95% CI) | 1.21 (0.87–1.69), *P*=0.25 | Ref. | 0.99 (0.71–1.39), *P*= 0.98 |  | 1.40 (0.97–2.01), *P*=0.06 | Ref. | 1.05 (0.73–1.52), *P*= 0.77 |
| ***Ischaemic stroke*** | |  |  |  |  |  |  |
| Model 1 (HR, 95% CI) | 1.17 (0.75–1.83), *P*=0.49 | Ref. | 0.97 (0.62–1.51), *P*=0.89 |  | 1.32 (0.80–2.16), *P*=0.27 | Ref. | 1.15 (0.70–1.87), *P*= 0.58 |
| Model 3 (HR, 95% CI) | 1.09 (0.69–1.70), *P*=0.71 | Ref. | 0.93 (0.59–1.46), *P*=0.76 |  | 1.21 (0.73–1.98), *P*=0.46 | Ref. | 1.09 (0.67–1.77), *P*= 0.74 |
| ***Haemorrhagic stroke*** |  |  |  |  |  |  |  |
| Model 1 (HR, 95% CI) | 1,42 (0.75–2.68), *P*=0.28 | Ref. | 1.05 (0.55–2.02), *P* = 0.87 |  | 2.27 (1.06–4.84), *P*=0.03 | Ref. | 1.32 (0.61–2.88), *P*= 0.48 |
| Model 3 (HR, 95% CI) | 1.37 (0.72–2.59), *P*=0.33 | Ref. | 0.99 (0.52–1.90), *P* = 0.98 |  | 2.09 (0.98–4.46), *P*=0.06 | Ref. | 1.21 (0.55–2.63), *P*= 0.64 |
| ***Unclassified stroke*** | |  |  |  |  |  |  |
| Model 1 (HR, 95% CI) | 1.57 (0.74–3.35), *P*=0.24 | Ref. | 1.22 (0.57–2.62), *P*=0.60 |  | 1.43 (0.68–3.02), *P*=0.35 | Ref. | 0.90 (0.42–1.94), *P*= 0.79 |
| Model 3 (HR, 95% CI) | 1.47 (0.69–3.15), *P*=0.31 | Ref. | 1.18 (0.55–2.54), *P*=0.67 |  | 1.34 (0.63–2.84), *P*=0.45 | Ref. | 0.86 (0.40–1.87), *P*= 0.71 |
|  | Loss  (<−10%) | Stable  (−10% –10%) | Gain  (>10%) |  | Loss  (<−10%) | Stable  (−10% –10%) | Gain  (>10%) |
| ***All stroke*** |  |  |  |  |  |  |  |
| Model 1 (HR, 95% CI) | 1.35 (1.02–1.78), *P*=0.03 | Ref. | 0.99 (0.75–1.32), *P*= 0.99 |  | 1.34 (1.01–1.78), *P*=0.04 | Ref. | 0.94 (0.70–1.25), *P*= 0.65 |
| Model 3 (HR, 95% CI) | 1.21 (0.91–1.59), *P*=0.19 | Ref. | 0.93 (0.70–1.23), *P*= 0.60 |  | 1.17 (0.88–1.56), *P*=0.27 | Ref. | 0.87 (0.65–1.16), *P*= 0.35 |
| ***Ischaemic stroke*** |  |  |  |  |  |  |  |
| Model 1 (HR, 95% CI) | 1.33 (0.91–1.95), *P*=0.15 | Ref. | 0.99 (0.67–1.46), *P*=0.95 |  | 1.14 (0.76–1.70), *P*=0.52 | Ref. | 0.93 (0.63–1.37), *P*= 0.70 |
| Model 3 (HR, 95% CI) | 1.19 (0.81–1.76), *P*=0.36 | Ref. | 0.92 (0.62–1.36), *P*=0.67 |  | 0.99 (0.66–1.48), *P*=0.95 | Ref. | 0.86 (0.58–1.27), *P*= 0.44 |
| ***Haemorrhagic stroke*** |  |  |  |  |  |  |  |
| Model 1 (HR, 95% CI) | 1.38 (0.82–2.33), *P*=0.23 | Ref. | 0.90 (0.52–1.57), *P*=0.72 |  | 1.98 (1.23–3.47), *P*=0.02 | Ref. | 1.12 (0.62–2.02), *P*= 0.71 |
| Model 3 (HR, 95% CI) | 1.24 (0.73–2.11), *P*=0.42 | Ref. | 0.81 (0.46–1.41), *P*=0.46 |  | 1.80 (1.02–3.16), *P*=0.04 | Ref. | 1.03 (0.57–1.86), *P*= 0.93 |
| Unclassified stroke |  |  |  |  |  |  |  |
| Model 1 (HR, 95% CI) | 1.37 (0.74–2.55), *P*=0.32 | Ref. | 1.15 (0.62–2.13), *P*=0.65 |  | 1.22 (0.67–2.24), *P*=0.52 | Ref. | 0.78 (0.41–1.45), *P*= 0.43 |
| Model 3 (HR, 95% CI) | 1.22 (0.65–2.28), *P*=0.53 | Ref. | 1.08 (0.58–1.99), *P*=0.81 |  | 1.09 (0.59–2.00), *P*=0.79 | Ref. | 0.73 (0.39–1.38), *P*= 0.34 |
|  | Loss  (<−20%) | Stable  (−20% –20%) | Gain  (>20%) |  | Loss  (<−20%) | Stable  (−20% –20%) | Gain  (>20%) |
| ***All stroke*** |  |  |  |  |  |  |  |
| Model 1 (HR, 95% CI) | 1.26 (0.91–1.75), *P*=0.16 | Ref. | 0.92 (0.68–1.25), *P*= 0.59 |  | 1.43 (1.07–1.91), *P*=0.01 | Ref. | 0.96 (0.72–1.27), *P*= 0.78 |
| Model 3 (HR, 95% CI) | 1.13 (0.81–1.57), *P*=0.47 | Ref. | 0.88 (0.65–1.19), *P*= 0.39 |  | 1.33 (0.99–1.79), *P*=0.05 | Ref. | 0.93 (0.70–1.23), *P*= 0.62 |
| ***Ischaemic stroke*** |  |  |  |  |  |  |  |
| Model 1 (HR, 95% CI) | 1.42 (0.91–2.22), *P*=0.12 | Ref. | 1.17 (0.79–1.74), *P*= 0.44 |  | 1.48 (0.99–2.23), *P*=0.05 | Ref. | 1.09 (0.75–1.60), *P*= 0.65 |
| Model 3 (HR, 95% CI) | 1.32 (0.84–2.07), *P*=0.23 | Ref. | 1.13 (0.76–1.68), *P*= 0.55 |  | 1.42 (0.94–2.14), *P*=0.09 | Ref. | 1.06 (0.73–1.56), *P*= 0.75 |
| ***Haemorrhagic stroke*** |  |  |  |  |  |  |  |
| Model 1 (HR, 95% CI) | 1.16 (0.62–2.15), *P*=0.65 | Ref. | 0.53 (0.26–1.07), *P*= 0.07 |  | 1.61 (0.95–2.73), *P*=0.09 | Ref. | 0.75 (0.41–1.34), *P*= 0.33 |
| Model 3 (HR, 95% CI) | 1.02 (0.55–1.92), *P*=0.94 | Ref. | 0.47 (0.23–0.95), *P*= 0.03 |  | 1.54 (0.90–2.62), *P*=0.11 | Ref. | 0.71 (0.39–1.27), *P*= 0.25 |
| ***Unclassified stroke*** |  |  |  |  |  |  |  |
| Model 1 (HR, 95% CI) | 1.06 (0.50–2.26), *P*=0.87 | Ref. | 0.89 (0.46–1.73), *P*= 0.74 |  | 1.11 (0.56–2.19), *P*=0.76 | Ref. | 0.93 (0.51–1.71), *P*= 0.82 |
| Model 3 (HR, 95% CI) | 0.89 (0.41–1.94), *P*=0.77 | Ref. | 0.83 (0.43–1.62), *P*= 0.59 |  | 0.99 (0.50–1.99), *P*=0.99 | Ref. | 0.90 (0.49–1.65), *P*= 0.73 |

PWR: platelet-to -white blood cell ratio; PNR: platelet-to-neutrophil ratio; hs-CRP: high-sensitivity C-reactive protein; Ref: reference; model 1: a crude hazard ratio model without adjustments; model 3: a multivariate model adjusted for age, sex, diabetes, hypertension, dyslipidaemia, smoking, alcohol consumption, physical activity, body mass index, self-rated health, cancer, genitourinary disease(nephropathy, prostatic disease, and gynecologic diseases) and chest disease(COPD, chronic bronchitis, emphysema, asthma, tuberculosis, and pneumonia), platelet count, and hs-CRP.
